# Predicting the Potential Impact of Emergency on Global Grain Security: A Case of the Russia–Ukraine Conflict

**DOI:** 10.3390/foods12132557

**Published:** 2023-06-30

**Authors:** Yuan Xu, Zhongxiu Wang, Wenjie Dong, Jieming Chou

**Affiliations:** 1 Key Laboratory of Environmental Change and Natural Disaster, MOE, Beijing Normal University, Beijing 100875, China; xuyuan01@mail.bnu.edu.cn (Y.X.); choujm@bnu.edu.cn (J.C.); 2 Institute of Disaster Risk Science, Faculty of Geographical Science, Beijing Normal University, Beijing 100875, China; 3 Chinese Academy of Fiscal Sciences, Beijing 100142, China; 4 Institute of Tibetan Plateau Research, Chinese Academy of Sciences, Beijing 100101, China; 5 Alliance of International Science Organizations, Beijing 100101, China; 6 School of Atmospheric Sciences, Sun Yat-sen University, Zhuhai 519082, China; dongwj3@mail.sysu.edu.cn

**Keywords:** emergency, Russia–Ukraine conflict, food security, sustainable development, composite index, ARIMA model

## Abstract

Global emergencies have a profound impact on exacerbating food insecurity, and the protracted Russia–Ukraine conflict has emerged as a significant driver of a global food crisis. Accurately quantifying the impact of this conflict is crucial for achieving sustainable development goals. The multi-indicator comprehensive evaluation approach was used to construct a grain security composite index (GSCI). Moreover, econometric model was used to predict the potential impacts of the conflict on global grain security in 2030 under two scenarios: with and without the “Russia-Ukraine conflict”. The results conclude that global food prices reached unprecedented levels as a consequence of the conflict, leading to notable fluctuations in food prices, especially with a significant surge in wheat prices. The conflict had a negative impact on global grain security, resulting in a decline in grain security from 0.538 to 0.419. Predictions indicate that the influence of the conflict on global grain security will be substantially greater compared to the scenario without the conflict in 2023–2030, ranging from 0.033 to 0.13. Furthermore, grain security will first decrease and then increase under the sustained consequences of the conflict. The achievement of the 2030 sustainable development goals will encounter significant challenges in light of these circumstances.

## 1. Introduction

The compounded impacts of global climate change have magnified the significance of global major emergencies as primary catalysts for worsening food insecurity. These emergencies exert substantial influence on global economic development, social stability, and the attainment of sustainable development objectives. Conducting quantitative analyses to assess the effects of these emergencies on food security is paramount for mitigating risks and strengthening resilience. In recent years, there have been an abundance of international reports addressing the global food crisis, including the “Global Report on Food Crises” [1], “The State of Food Security and Nutrition in the World” [2], “Global Food Security Index” [3], and “Global Hunger Index” [4]. Since the turn of the 21st century, the global food system has experienced recurrent major emergencies characterized by sharp increases in food prices. These emergencies encompass the 2008 economic crisis [5], the 2011 Syrian civil war [6,7,8], the 2019 COVID-19 pandemic [9], and the 2022 Russia–Ukraine conflict [10,11]. Adverse phenomena such as diminishing crop production, labor shortages, soaring food prices, inadequate food supply, limited food accessibility, and disruptions in trade have been ubiquitously observed [12,13,14]. It is projected that by 2030, more than 8% of the world’s population (approximately 670 million people) will confront the issue of hunger [2]. Hence, conducting quantitative analyses on the repercussions of emergencies, exemplified by the Russia–Ukraine conflict, on global food security is imperative for effectively addressing the resultant damages and risks.

Since the outbreak of the Russia–Ukraine conflict in February 2022, global food production has been reduced, food prices have exceeded historical highs, and food layout has changed [10]. Predicting future changes in world food security trends is of great significance for achieving UN sustainable development goals (SDGs). With the escalating conflict between the Russian Federation (Russia) and Ukraine, the dominant position of the two countries in the global food landscape has been gradually weakened, and the global food security situation has been deteriorating [11,15,16,17]. Countries that are highly dependent on Russia and Ukraine are at risk of food shortages, and the international community generally believes that a global food crisis has occurred [2,13,14]. The conflict, combined with other factors, will perpetuate global food insecurity and will have a profound impact on the future development of the world order, making food security an urgent issue for current global governance.

Food security is a complex and multidimensional issue which encompasses various dimensions, factors, and levels [18,19,20]. Globally, numerous scholars and international organizations have developed different food security indicators and composite indices, including the Global Hunger Index (GHI) [21], the Global Food Security Index (GFSI) [3], and a comprehensive set of food security indicators [22]. Composite indices serve not only to summarize intricate and multidimensional concerns but also to facilitate a more accessible interpretation of the overall characteristics of complex issues, surpassing the analysis of individual indicators [23]. Empirical analysis based on multidimensional and multi-indicator composite indices has proven to be a valuable tool for measuring food security at both regional and national levels. Currently, there exists a wealth of insightful perspectives from domestic and international sources regarding the impact of the Russia–Ukraine conflict on food security, Notably, influential studies conducted by Ben Hassen and El Bilali [12], Nasir et al. [16], and Jagtap et al. [24] have extensively reviewed the literature to provide a qualitative understanding of the consequences of this conflict on food security. Their investigations have uncovered substantial short-term and long-term implications, including disruptions in global supply chains and food trade, destabilization of markets, sharp increases in food prices, and significant threats to the attainment of SDGs. It is important to note, however, that these studies have predominantly relied on individual indicators such as food prices [25,26] and food production [11,24] to evaluate the overall state of food security, thus offering a qualitative depiction of the impact of the Russia–Ukraine conflict on food security. However, it is important to recognize that food security is not determined by a single factor or indicator but rather represents a comprehensive reflection of multiple dimensions and indicators [22,27,28,29]. Currently, there is a dearth of quantitative assessment and future trend prediction concerning the multidimensional and multi-indicator impacts on food security. To bolster resilience and the ability to withstand present and future shocks stemming from emergencies, it is imperative to construct a composite index that captures the multifaceted nature of food security from a multidimensional and multi-indicator perspective. The optimization of parameters and the improvement of accuracy in assessing the impacts of food security hold great significance.

Hence, this study encompasses a global perspective and selects the Russia–Ukraine conflict as a representative case of a significant emergency. Firstly, building upon FAO’s multidimensional theoretical framework of food security, this paper constructs a grain security evaluation indicator system and applies the multi-indicator comprehensive evaluation approach to construct a grain security composite index (GSCI). Secondly, the autoregressive integrated moving average (ARIMA) model is used to predict the potential effects on global grain security in 2030, encompassing both scenarios with and without the Russia–Ukraine conflict.

## 2. Construction of the Grain Security Evaluation Indicator System

Food security has four dimensions: availability, access, stability, and utilization, which means that a situation that exists when all people, at all times, have physical, social and economic access to sufficient, safe, and nutritious food that meets their dietary needs and food preferences for an active and healthy life [22,30]. The understanding of food security varies across different scales and dimensions. It emphasizes not only the macro level of national or global grain supply and access but also the micro level of dietary needs to maintain the health and active life of families or individuals [31,32]. The macro-scale understanding of food security primarily revolves around the global, regional, or national levels, with a key research objective of ensuring an adequate food supply and enabling access to food through sufficient income or resources [32,33]. This perspective places significant emphasis on the availability and access dimensions of food security. Notably, the Economist Intelligence Unit (EIU) developed the Global Food Security Index (GFSI) in 2012, which integrated 28 indicators pertaining to the affordability, availability, quality, and safety of food [34,35]. The GFSI served as an assessment tool for evaluating the current state of food security at the national level [20]. In 2013, the Food and Agriculture Organization (FAO) introduced a comprehensive set of 30 food security indicators encompassing dimensions related to the availability, access, stability, and utilization of food [22]. These indicators were instrumental in monitoring countries’ progress toward achieving global food security goals.

At the micro-scale, food security is primarily concerned with household or individual levels, aiming to evaluate the utilization of food for nutrient extraction and ensure an adequate intake of calories and dietary energy [36,37]. This perspective highlights the utilization dimension of food security. The concept of “nutritional security” has emerged as a deeper understanding of food security, encompassing micro-level factors such as dietary energy, care practices, health and sanitation conditions, micronutrients, and nutritional components [21,22], thus providing a focused reflection of the utilization dimension. The macro-level food security to some extent determines the nutritional security at the micro-level, as the achievement of nutritional security and food security at the household or individual levels relies on sufficient food supply and accessibility at the national or regional level [31,32]. For example, the Global Hunger Index (GHI) developed by the International Food Policy Research Institute (IFPRI) utilized three indicators: the proportion of undernourished population, the prevalence of underweight in children, and the child mortality rate under the age of five. The GHI served as an assessment tool for evaluating hunger and nutritional security at the household or individual levels [21].

The diverse scales of food security display distinct and dynamic processes of change. A comprehensive assessment that combines these scales would obscure the unique trends and characteristics inherent to each scale. Hence, it is essential to concentrate on a specific scale and investigate the patterns of change within a particular domain to propose scientifically effective response measures. Accordingly, this study focuses on the macro-level of food security and does not consider the micro-level aspect of nutrition security. To differentiate between food security and nutrition security, the macro-level of food security is defined as grain security, while the micro-level is defined as nutrition security. Consequently, food security encompasses both macro-level grain security and micro-level nutrition security. Drawing from the food security evaluation framework and indicator system established by FAO (Appendix A), indicators are selected from the dimensions of availability, access, and stability to construct the macro-level grain security evaluation indicator system (Table 1).

## 3. Materials and Methods

### 3.1. Study Area

**Figure 1:**
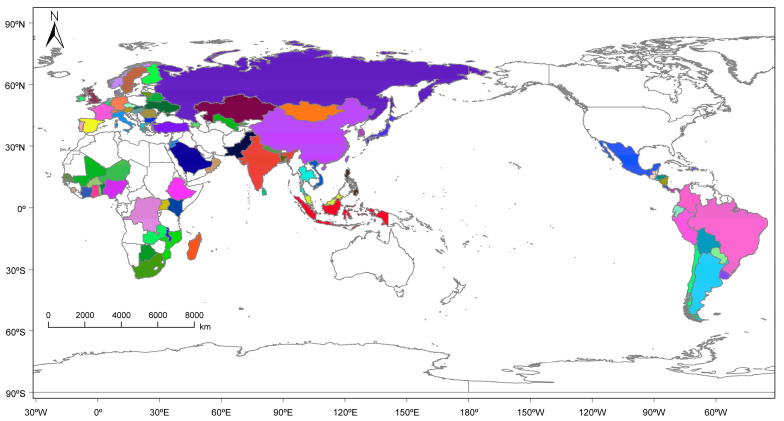
Study area (Countries with colors are the study area of this paper.).

This study focuses on a global scale, taking into account the standards used by FAO to analyze global food security and nutritional status [2]. Based on the availability and completeness of data for grain security indicators across different countries, a selection of 86 countries was made (Table 2), as shown in Figure 1. The global-scale values represented the average of the indicator values across all countries.

### 3.2. Simulation of the Grain Security Composite Index Based on the Multi-Indicator Comprehensive Evaluation Method

Grain security is a comprehensive variable with multiple dimensions and indicators. Constructing a composite index for grain security allows for a comprehensive reflection of complex and multi-faceted issues, as well as the identification of overall trends to explain complex problems [23]. Currently, both domestic and international approaches often utilize a combination of multiple indicators weighted together to represent the overall characteristics of grain security [22,33,38]. Therefore, this study employs the multi-indicator comprehensive evaluation method to construct a grain security composite index (GSCI). The multi-indicator comprehensive evaluation method involves four main steps: data normalization, determination of indicator weights, construction of evaluation models, and simulation of the composite index [29,33]. The specific steps of the method are as follows:

**(1) Data normalization**: the range method is used to normalize the indicator data. Equation (1) is applied to indicators expected to have a positive impact on grain security, while Equation (2) is used for indicators expected to have a negative impact. Where $X_{ij}$ represents the raw data of the *j*-th indicator in the *i*-th region, $X_{ij}^{\prime }$ $X_{j}$ and min$X_{j}$ represent the maximum and minimum values of the *j*-th indicator, respectively. Normalized data are available in the Supplementary Materials.

(1) $$X_{ij}^{\prime }=\left(X_{ij}-minX_{j}\right)/\left(maxX_{j}-minX_{j}\right),$$

(2) $$X_{ij}^{\prime }=\left(maxX_{j}-X_{ij}\right)/\left(maxX_{j}-minX_{j}\right),$$

**(2) Indicator weight determination:** this study combines the subjective Analytic Hierarchy Process (AHP) and the objective Criteria Importance Though Intercriteria Correlation (CRITIC) method, aiming to consider both the subjective judgment of decision-makers and the objective characteristics of the evaluation objects. The AHP method subjectively decomposes the evaluation objectives into different hierarchies and indicators, comparing and calculating the indicators within the same hierarchy to determine their weights [39,40]. Through the literature review and based on existing research results [3,29,35,41,42,43,44], pairwise comparisons were conducted for the indicators of grain security to construct the pairwise comparison matrix. The geometric mean method was then used to calculate the weights of each indicator. The calculation formula is shown in Equation (3). In Equation (3), $C_{ij}$ represents the matrix elements, and $w_{i}$ represents the subjective weight value of the *i*-th indicator. The CRITIC method objectively calculates the weights of indicators based on the intensity of comparison and conflicts between indicators [45,46]. The intensity of comparison is represented by the standard deviation of indicator data, measuring the magnitude of fluctuations in the indicator data. The conflicts between indicators are represented by the correlation coefficients of the indicator data, measuring the interrelationships between indicators. The calculation formula is shown in Equation (4). In Equation (4), $w_{j}$ is the weight of indicator *j*, $r_{ij}$ is the correlation coefficient between indicators *i* and *j*, and $\sigma_{j}$ is the standard deviation of indicator *j*. Finally, the subjective weights calculated by the AHP method and the objective weights calculated by the CRITIC method are combined to obtain comprehensive weights [47]. The calculation formula is shown in Equation (5). In Equation (5), $\omega_{i}$ represents the subjective weight value obtained by the AHP method, $\omega_{j}$ represents the objective weight value obtained by the CRITIC method, and $\omega_{ij}$ represents the comprehensive weight value of the indicator. The calculated values of each indicator of grain security based on the mixed weighting method are shown in Table 3.

(3) $$w_{i}=\frac{\sqrt[{n}]{\prod_{j=1}^{n}C_{ij}}}{\sum_{j=1}^{n}\sqrt[{n}]{\prod_{j=1}^{n}C_{ij}}},$$

(4) $$w_{j}=\frac{\sigma_{j}\sum_{i=1}^{n}\left(1-r_{ij}\right)}{\sum_{j=1}^{m}\sigma_{j}\sum_{i=1}^{n}\left(1-r_{ij}\right)},$$

(5) $$\omega_{ij}=\frac{\omega_{i}\omega_{j}}{\sum \omega_{i}\omega_{j}}$$

**(3) Construction of the evaluation model and composite index:** based on the normalized dataset and indicator weights, a grain security evaluation model is developed, as described by Equations (6) and (7). The second-level index layer evaluation model, represented by Equation (6), is utilized for assessing grain’s availability ($Y_{1}$), access ($Y_{2}$), and stability ($Y_{3}$). The first-layer indicator layer evaluation model, represented by Equation (7), is employed for the evaluation of GSCI, which serves as a comprehensive measure of grain security. The data of GSCIs are available in the Supplementary Materials.

(6) $$Y_{it}=\sum \left(\omega_{ij}\cdot X_{ijt}\right),$$

(7) $$GSCI_{t}=\sum Y_{it},$$

### 3.3. ARIMA Prediction Model

The ARIMA model is used to predict global GSCI in the future. The autoregressive integrated moving average (ARIMA) model is a commonly used model in time series analysis and prediction methods [48,49]. The model is widely used to analyse time series data in various fields [50,51,52]. The model is based on the law and past and present historical data to estimate and infer the state of something at some point in the future [53,54]. The model consists of three parts: the autoregressive model (AR model), the differential and the moving average model (MA model), denoted as ARIMA (*p*, *d*, *q*), where *p*, *d* and *q* represent the order of the autoregressive, differential and moving average, respectively. Using this model for prediction generally involves a data unit root test and stationary processing, model identification, model parameter estimation, and testing steps [52,54,55]. In the ARIMA model, the future value of the sequence is expressed as a linear function of the current and lag periods of the lag term and the random disturbance term. That is, the general form of the model is as follows:

(8) $$\hat{G_{t}}=\mu +\alpha_{1}G_{t-1}+\alpha_{2}G_{t-2}+\ldots +\alpha_{p}G_{t-p}+\varepsilon_{t}+\beta_{1}\varepsilon_{t-1}+\beta_{2}\varepsilon_{t-2}+\ldots +\beta_{q}\varepsilon_{t-q},$$

where $\hat{G_{t}}$ represents the predicted value of the model. $G_{t}$ represents the measured value of the original sequence. $\alpha_{1},\alpha_{2}\ldots \alpha_{p}$ is the coefficient of the AR model, and *p* is the order of the AR model. $\beta_{1},\beta_{2}\ldots \beta_{q}$ denote the coefficients of the MA model, and *q* denotes the order of the MA model. μ denotes a constant and $\varepsilon_{t}$ represents a white noise process. The optimal ARIMA (*p, d, q*) model is determined by analyzing the autocorrelation function (ACF) and partial autocorrelation function (PACF). The appropriate values for *p*, *d*, and *q* are selected using the Akaike Information Criterion (AIC) and Bayesian Information Criterion (BIC). When the sample size *N* is fixed, the values of *p* and *q* are determined by selecting the minimum AIC and BIC values. The equations are as follows:

(9) $$AIC\left(p,q\right)=Nln\sigma^{2}\left(p,q\right)+2\left(p+q+1\right),$$

(10) $$ABIC\left(p,q\right)=Nln\sigma^{2}\left(p,q\right)+\left(p+q+1\right)lnN,$$

To test the validity of the ARIMA forecasting model, this study employs several test metrics, including Root Mean Square Error (RMSE), Mean Absolute Percentage Error (MAPE), and the coefficient of determination R^2^ [52,54,56]. A smaller RMSE and MAPE indicate a closer proximity between the predicted values and the observed values, with values closer to 0 being desirable.

(11) $$RMAE=\sqrt{{\left(G_{t}-\hat{G_{t}}\right)}^{2}},$$

(12) $$MAPE=\overset{N}{\underset{i=1}{\sum }}\left|\frac{G_{t}-\hat{G_{t}}}{G_{t}}\right|\times \frac{100}{N},$$

### 3.4. Scenario Assumptions of the Russia–Ukraine Conflict

To quantify the impact of the conflict between Russia and Ukraine on global grain security, this study aims to assess the effects of the conflict by analyzing the differences in the developmental status of grain security under two scenarios “with” and “without” the conflict.

Considering the conflict, which commenced in February 2022, as a case study, we define *Y* as the grain security composite index (GSCI), *E* as the status of the Russia–Ukraine (R–U) conflict, and $X_{i}$ as the set of other influencing factors, such as climate change, production conditions, technological advancements, and policy systems. A functional relationship exists between the GSCI and its influencing factors [20,28,33,57], expressed as $Y=f\left(E,\;X_{i}\right)$. This study assumes two scenarios: scenario without the R–U conflict and scenario with the R–U conflict. The former is an idealized state, and the latter is an actual state.

**Figure 2:**
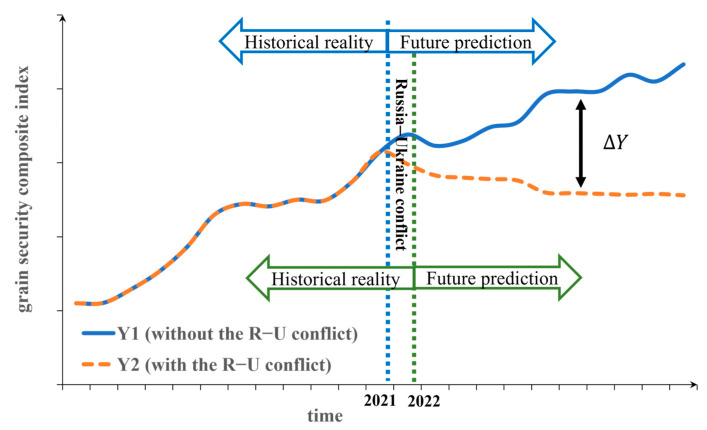
Changes in the grain security composite index under the two scenarios without and with the Russia–Ukraine conflict (Y1 represents the changes in the GSCI without the R–U conflict scenario (idealized state), Y2 represents the changes in the GSCI with the R–U conflict scenario (actual state)).

The scenario without the R–U conflict refers to the fact that grain security still changes based on the time series before the Russia–Ukraine conflict. The data from 2001 to 2021 are used to predict global grain security in 2022–2030, which is recorded as $Y_{1}$. The scenario with R–U conflict refers to the change in grain security based on the time series changes after the R–U conflict has occurred. The data from 2001 to 2022 are used to predict global grain security in 2023–2030, which is recorded as $Y_{2}$. The difference between $Y_{2}$ and $Y_{1}$ is due to the influence of the R–U conflict, that is $∆Y=Y_{2}-Y_{1}$ (Table 4 and Figure 2). Therefore, by comparing and analyzing the average level of global grain security predicted under the two scenarios without and with the R–U conflict, the purpose of quantitatively distinguishing the impact of the R–U conflict is achieved.

### 3.5. Data Source and Preprocessing

The data comprise grain security indicators from 86 countries spanning the years 2001 to 2022. It encompasses a total of 14 indicators. The primary data sources include the Food and Agriculture Organization of the United Nations (FAO) through FAOSTAT (https://www.fao.org/faostat/zh/#data (accessed on 12 April 2022)), the World Bank (https://databank.worldbank.org/source/world-development-indicators (accessed on 12 April 2022)), the United Nations Commodity Trade Statistics Database (UN Comtrade Database) (https://comtrade.un.org/ (accessed on 12 April 2022)), and the Agricultural Market Information System (AMIS) (https://www.amis-outlook.org/home/en/ (accessed on 12 April 2022)). Specific details of the indicators are presented in Table 1. Following the data statistical principles of FAO and to eliminate abnormal trend fluctuations, all data underwent a three-year moving average preprocessing. Linear interpolation is employed to fill in missing values in the dataset. Global values represent the average of indicator values across all countries.

## 4. Results

### 4.1. Current Status of Grain Security in Russia and Ukraine

#### 4.1.1. Current Status of Grain Production

**Figure 3:**
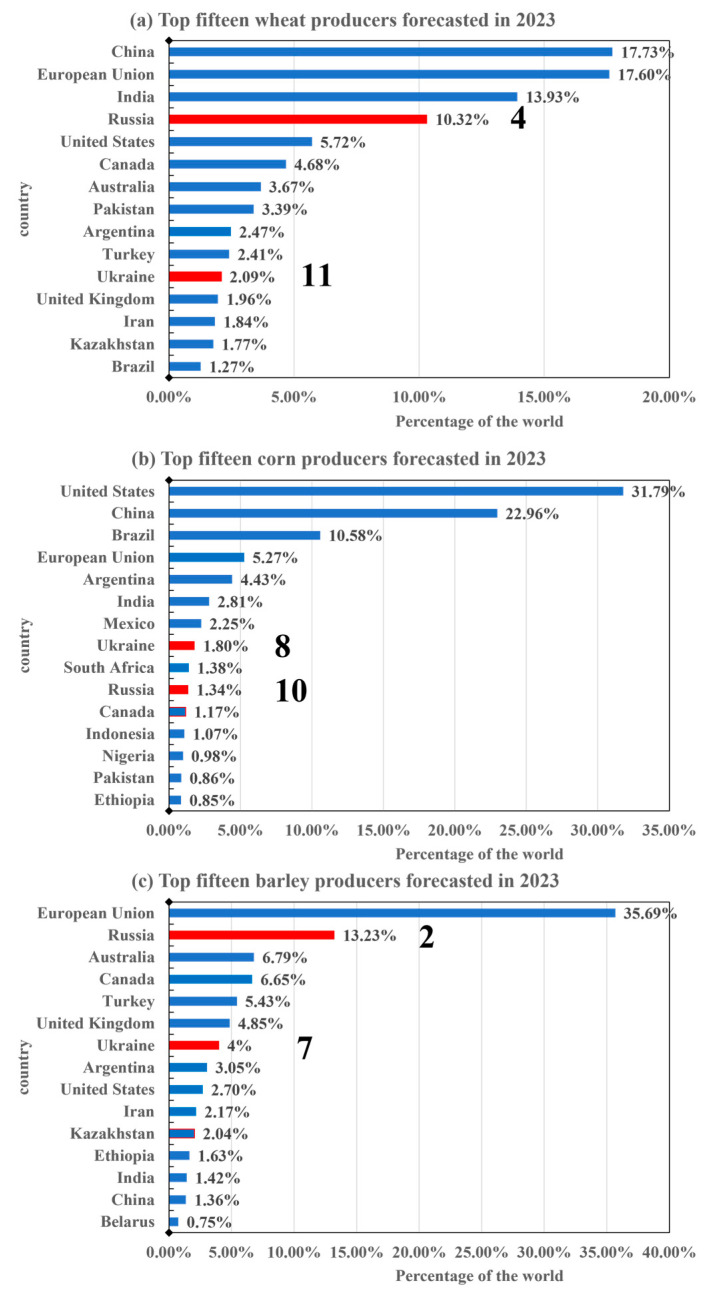
Top 15 countries in crop production forecasted in 2023.

Russia and Ukraine are the world’s major food producers, and furthermore, have superior geographical and climatic conditions for food production [58]. It is forecasted that by 2023, Russia is the world’s fourth largest wheat producer after China, the European Union, and India, accounting for 10.32% of global wheat production. Meanwhile, Ukraine ranks 11th, with 2.09% of global wheat production (Figure 3a). Russia and Ukraine are the tenth and eighth largest corn producers in the world, respectively. It is estimated that by 2023, Russia’s and Ukraine’s corn production will account for 1.34% and 1.8% of global corn production, respectively (Figure 3b). For barley, Russia ranks as the second largest producer globally, following the European Union. In 2023, Russia accounted for 13.23% of global barley production, while Ukraine ranked seventh, contributing to 4% of global barley production in the same year (Figure 3c). These numbers show that Russia and Ukraine are the most important food producers in the world and play an important role in the global food supply chain [24].

#### 4.1.2. Current Status of Grain Trade

**Figure 4:**
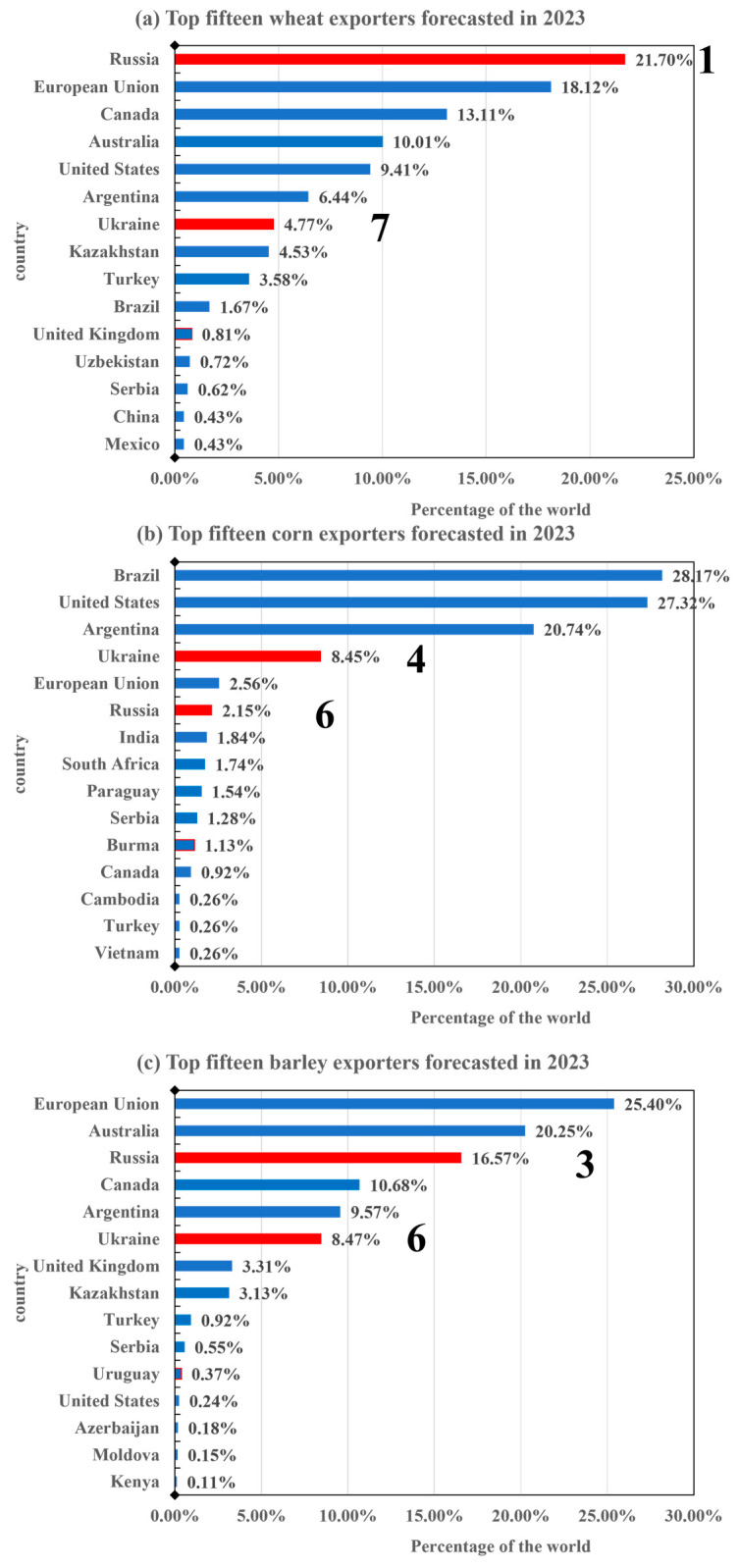
Top 15 countries in crop exports forecasted in 2023.

Russia and Ukraine are not only major grain producers but are also major grain exporters, which is related to the relatively limited population and consumption of the two countries [58]. The two countries have high crop yields, especially for grain, but low consumption. They thus can use more goods for exporting. Russia is the world’s largest wheat exporter, accounting for approximately one-fifth of global exports, according to 2023 forecasts. Meanwhile, Ukraine ranks seventh, with 4.77% of global wheat exports (Figure 4a). In addition, Ukraine and Russia are the world’s fourth and sixth largest corn exporters, respectively, and their corn exports are expected to account for 8.45% and 2.15% of global corn exports by 2023 (Figure 4b). Russia accounts for 16.57% of global barley exports, making it the third largest barley exporting country worldwide, following the European Union and Australia. Ukraine, on the other hand, ranks sixth, with its barley exports contributing to 8.47% of the global export volume (Figure 4c).

#### 4.1.3. Current Status of Food Prices

**Figure 5:**
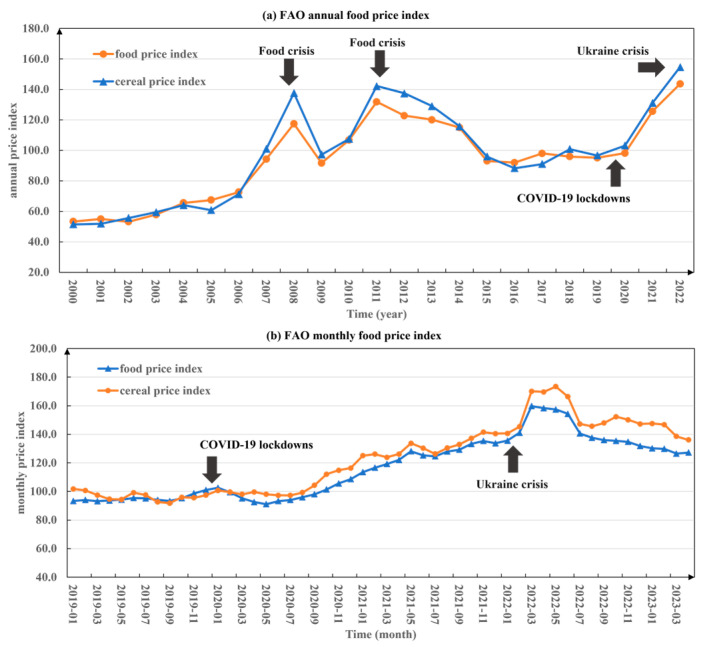
Global food and cereal prices trends since the 21st century (2000–2022) ((**a**) depicts the annual variations in the food price index and the cereal price index from 2000 to 2022. (**b**) illustrates the monthly changes from January 2019 to April 2023. The data were sourced from the Food and Agriculture Organization (FAO).).

Since the 21st century, global food prices have experienced two obvious food crises in 2008 and 2011 under the continuous influence of climate variability and extreme weather, conflict, economic slowdown, and recession, all of which led to a sharp increase in food prices and a record high. Global food prices demonstrated a stable trajectory leading up to 2020; however, commencing from that year, they underwent a notable upswing owing to a multitude of factors, encompassing the repercussions of the COVID-19 pandemic. In 2022, global food prices once again reached record highs, surpassing the price peaks of 2008 and 2011, and the conflict between Russia and Ukraine is an additional driver of these record highs (Figure 5). The surge in global food price has raised concerns about the possibility and potential impact of another global food crisis, which may exacerbate hunger in poor areas as well as social unrest around the world and fluctuations in the global food market [59]. Russia and Ukraine are important players in the global trade in food and agricultural products, particularly wheat, corn, and barley. Several risks arising from the conflict will directly and indirectly affect global supply. Among them, the risk of disruption of trade flows and the resulting risk of soaring prices are the most important to consider.

**Figure 6:**
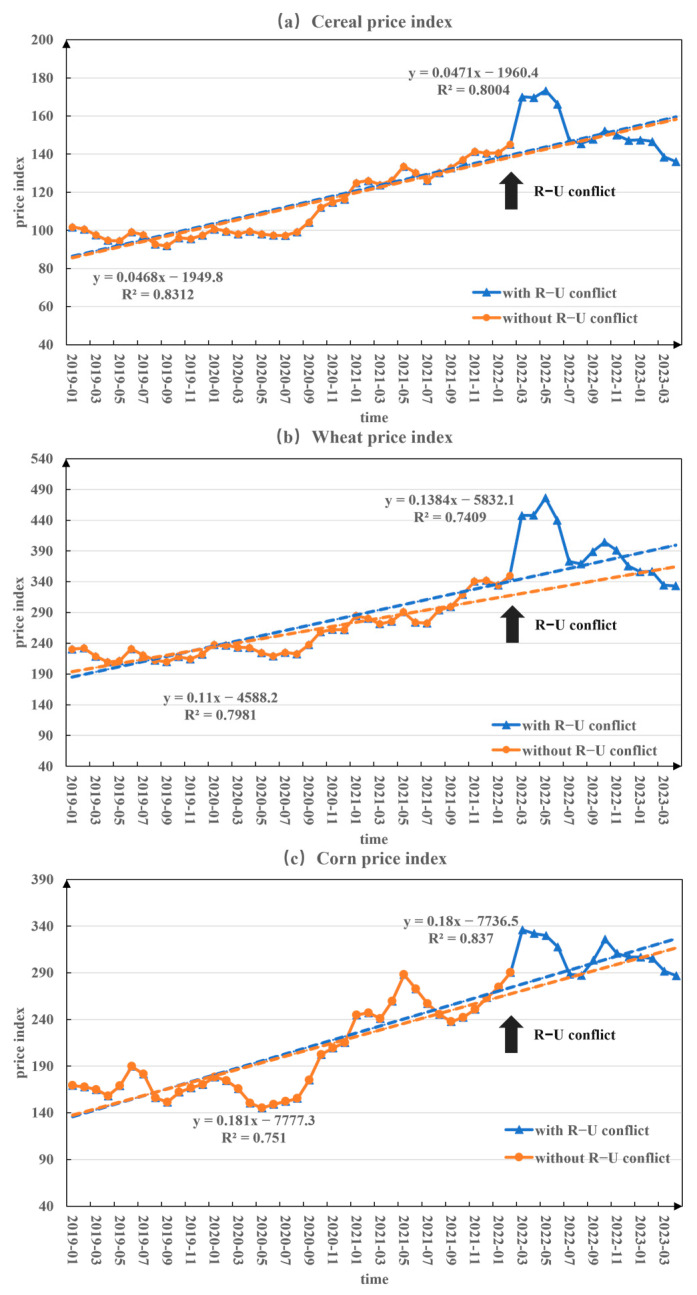
Monthly changes in price indices for global cereal, wheat, and corn during the period without and with the Russia–Ukraine conflict (the period without the R–U conflict is January 2019 to February 2022, and the period with the R–U conflict is January 2019 to April 2023).

To more clearly analyze the extent to which the conflict has affected food prices, this paper compares the evolution of global food prices during the period without the R–U conflict (January 2019 to February 2022) and with the R–U conflict (January 2019 to April 2023) based on monthly global food price data (Figure 6). During the period with the R–U conflict, the linear trend rates of global cereals, wheat, and corn prices were higher compared to the period without the conflict. This indicates that the Russia–Ukraine conflict stimulated an increase in prices of crops. Among them, the price of wheat experienced the greatest magnitude of change influenced by the conflict, increasing from 1.1/(10 mons) to 1.384/(10 mons), followed by corn. This may be because during the conflict, the cities and rural areas of the Ukraine state were severely damaged, and there was a shortage of agricultural labor. Ukraine’s land, crops, and agricultural infrastructure were damaged. Agricultural materials such as seeds and fertilizers were blocked due to the agricultural transport supply. For Russia, the severe sanctions of Western countries and their own anti-sanctions caused a double impact. In addition, financial and transportation restrictions may lead to difficulties in food sales, which in turn increases the shortage of food exports.

Regardless of whether the commodity is cereal, wheat, or corn, after February 2022, the price presents a fluctuating trend of first rising, then falling, and then rising again. This fluctuation in food prices may aggravate the uncertainty of the global grain market and cause unpredictable consequences for global food security. Results indicate that when food prices rise, people reduce their consumption of more expensive nutritious foods, such as fruits, vegetables, meat, and dairy. And they maintain calorie consumption by buying more processed foods or cheap staple foods, such as rice and maize [13,60]. This not only directly affects the quality of people’s diet, as higher food prices have a more serious impact on low-income countries [9].

### 4.2. Historical Assessment of the Impact of the Russia–Ukraine Conflict on Global Grain Security

**Figure 7:**
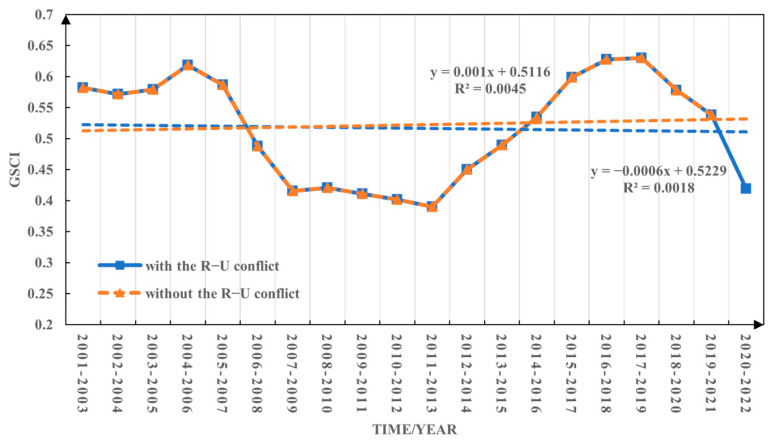
Changes in global grain security composite index during the period without the Russia–Ukraine conflict (2001–2021) and with the Russia–Ukraine conflict (2001–2022).

Based on the GSCI’s evaluation model, the global GSCI was calculated for the period without the R–U conflict (2001–2021) and the period with the R–U conflict (2001–2022). The GSCI demonstrated an upward trend with a growth rate of 0.01/(10a) during the period without the conflict (Figure 7). Conversely, during the period with the conflict, GSCI exhibited a downward trend with a rate of change of −0.006/(10a) (Figure 7). These observations unequivocally highlight the adverse impact of the Russia–Ukraine conflict on GSCI, implying its detrimental consequences for global grain security. Moreover, this effect manifests in a conspicuous transition from an ascending to a descending linear trend in the GSCI. In addition, global grain security has shown a fluctuating trend of first rising and then declining in the past 20 years. Among them, the period from 2005−2007 to 2007−2009 was in a rapid decline stage, mainly because during this period, the United States of America, the European Union, India, and other major grain-producing countries were affected by extreme weather events, resulting in frequent declines in wheat, maize, and other major grain crops [61]. In response to the continuing impact of the 2008 global financial crisis and to safeguard their own food security, some countries have introduced a series of trade policies to restrict agricultural product exports, which has led to an increase in global food insecurity. The period from 2019−2021 to 2020−2022 is also in a rapid decline stage. The main reason may be that the demand for quarantine testing brought by the COVID-19 pandemic has led to a blockage in food transportation and a reduction in food supply. The shift between supply and demand has led to rising food prices. The Russia–Ukraine conflict has led to the blockage of the food supply chain, seriously affecting the world market’s supply of major food commodities such as cereal, wheat and maize, thus causing the world to face a pattern of food insecurity [13].

### 4.3. Predicting the Potential Impact of the Russia–Ukraine Conflict on Global Grain Security in the Future

#### 4.3.1. Parameter Estimation and Validity Test of ARIMA Prediction Model

To investigate whether the sustained impact of the Russia–Ukraine conflict on global grain security would affect the achievement of the Sustainable Development Goals (SDGs) by 2030, this study utilizes an econometric modeling approach (ARIMA model) to predict the changes of GSCI under the scenarios of “without the R–U conflict” and “with the R–U conflict” for the future, 2030.

**Figure 8:**
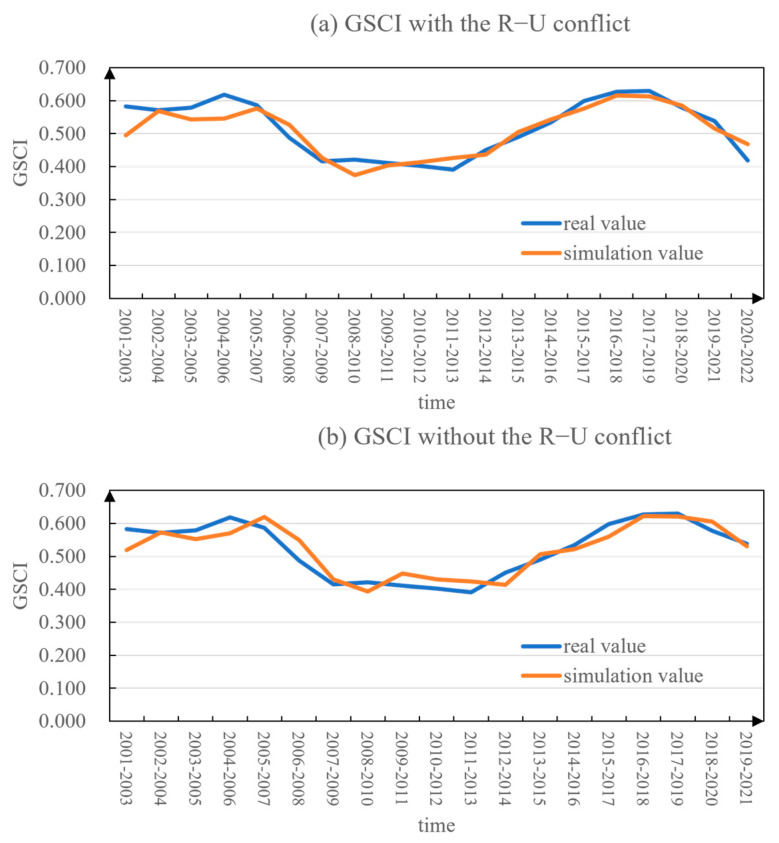
Simulation results of global grain security composite index from 2001 to 2022 under two scenarios (with or without the “Russia−Ukraine conflict”) based on ARIMA model.

For the scenario of “with the R–U conflict”, a unit root test is conducted on the global GSCI time series for the period 2001–2022 (Table 5). The results indicated that the data are a stationary series and passes the test at a significant level (*p*-value of 0.0). The autocorrelation plot showed that the autocorrelation coefficients significantly decrease after lag 2 and mostly remain within the 95% confidence interval (Appendix B). The partial autocorrelation function (PACF) plot revealed that the partial autocorrelation coefficients significantly decrease after lag 1 and mostly fall within the 95% confidence interval (Appendix B). Therefore, the ARIMA (2, 0, 1) model was considered the most suitable for this time series. By fitting the model through parameter estimation and conducting overall significance tests, the ARIMA model was obtained as shown in Equation (13). The goodness-of-fit of the model, represented by R^2^, was 0.826. The simulation results demonstrated a close alignment between the simulated values and the actual values (Figure 8a), with an average relative error of 4.73%. Furthermore, the model’s suitability was assessed utilizing the QQ plot and the normal distribution plot. As depicted in Appendix C, the scatter points displayed a concentration near the fitted line, and the residuals, representing the disparities between the actual and predicted values, adhered to a normal distribution. These observations suggest that the ARIMA (2, 0, 1) model was suitable for forecasting the GSCI trends with the Russia–Ukraine conflict scenario. Furthermore, the model’s predictive performance was evaluated employing two performance metrics: Root Mean Square Error (RMSE) and Mean Absolute Percentage Error (MAPE). The results revealed an RMSE value of 0.0347 and an MAPE value of 0.05232, both approaching zero, indicating a close correspondence between the predicted and actual values. Collectively, the ARIMA (2, 0, 1) model showcased a high degree of fitting accuracy and demonstrates robust predictive capabilities.

(13) $$G_{t}=0.495+1.724G_{t-1}-0.957G_{t-2}-\varepsilon_{t-1},$$

For the scenario of “without the R–U conflict”, the GSCI time series for the period 2001–2021 underwent a unit root test (Table 5). The results indicated that after applying a second-order difference, the time series became stationary and passed the significance level test (*p*-value is 0.006). Similarly, the autocorrelation and partial autocorrelation analyses (Appendix B) suggested that the most appropriate model for this series is ARIMA (2, 0, 0). By fitting the model using the estimated parameters and conducting overall significance tests, the ARIMA model is obtained as shown in Equation (14). The simulation results demonstrated a high level of agreement between simulated and actual values (Figure 8b), with an average relative error of 5.26%. The model’s goodness of fit, as indicated by the coefficient of determination (R^2^), was 0.84. Based on the scatter plot in Appendix C, it can be observed that the data points exhibit a close clustering around the fitted line, and the residuals, representing the differences between the actual and predicted values, demonstrate conformity to a normal distribution. These findings provided evidence supporting the suitability of the ARIMA (2, 0, 0) model for forecasting the trends in GSCI under the scenario without the conflict. Furthermore, the assessment of the model’s effectiveness indicated an RMSE value of 0.0328 and an MAPE value of 0.05541, both of which were in close proximity to 0. This suggested a high degree of concordance between the predicted and actual values. In summary, the ARIMA (2, 0, 0) model exhibited a commendable level of fitting accuracy and manifests favorable predictive performance.

(14) $$G_{t}=0.519+1.418G_{t-1}-0.657G_{t-2},$$

#### 4.3.2. Prediction of the Possible Impact of the Russia–Ukraine Conflict in 2030

**Figure 9:**
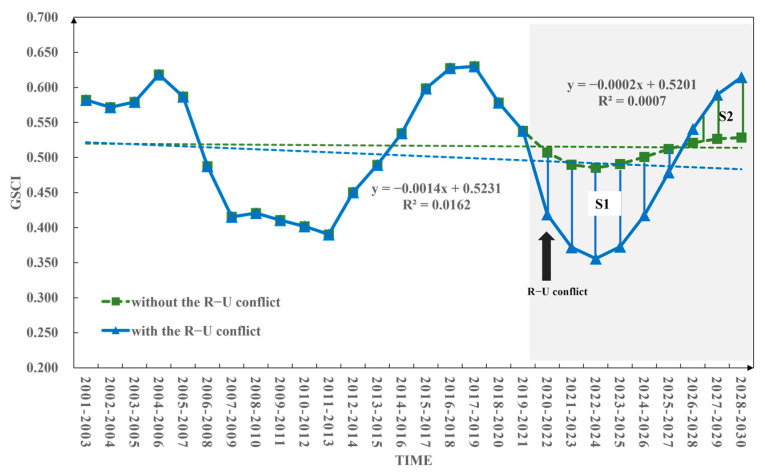
The prediction of global grain security composite index for 2030 under two scenarios (with or without the “Russia-Ukraine conflict”).

The validation of the ARIMA model’s simulation performance presented above demonstrates its feasibility in predicting the GSCI. Consequently, leveraging the GSCI data from 2001 to 2022 as the foundation, this study employs the ARIMA model to predict the GSCI under two scenarios: “with” and “without” the R–U conflict (Figure 9).

In both scenarios, the projected trends for GSCI from 2023 to 2030 will demonstrate an initial decline followed by an upward trend, with a trough observed during the period from 2022 to 2024, aligning with historical patterns of fluctuation (Figure 9). This pattern can be attributed to the ARIMA model’s assumption of stationarity based on long-term historical time series and its utilization of moving average extrapolation for future trend forecasting. Consequently, the anticipated trend changes are expected to replicate the overall fluctuation pattern of decline followed by ascent observed since 2001. Furthermore, it is worth noting that the magnitude of GSCI’s volatility under the scenario with the R–U conflict is significantly higher than that under the scenario without the conflict, further emphasizing the substantial impact of the Russia–Ukraine conflict on grain security dynamics.

From 2020–2022 to 2025–2027, the GSCI with the R–U conflict scenario will be lower than that without the R–U conflict scenario, with the difference shown in shadow part S1 in Figure 9. This shows that the R–U conflict will have a negative impact on global grain security, which will lead to a decline in grain security between 0.033 and 0.13. However, from 2026–2028 to 2028–2030, the GSCI with the R–U conflict scenario will be higher than that without the R–U conflict scenario. The difference is shown in shadow part S2 in Figure 9, and the degree of influence is between 0.02 and 0.086. It is evident from these two comparisons that the area of S1 is larger than that of S2, indicating that by 2030, future grain security will be significantly more affected by the conflict than not. Simultaneously, the research reveals that the Russia–Ukraine conflict will have both short-term and long-term effects on global grain security dynamics from 2023 to 2030. In the short term, the conflict will act as a primary driver leading to a decline in global grain security level, primarily exerting negative impacts. In the long term, there is a gradual recovery in global grain security level, which aligns with the historical fluctuations observed in the GSCI. This recovery can be attributed to the significant short-term impact caused by the Russia–Ukraine conflict, prompting countries to implement a series of preventive measures aimed at mitigating the losses arising from the conflict. Consequently, the long-term improvement in global grain security level is not solely driven by the Russia–Ukraine conflict but likely represents a comprehensive manifestation of the combined effects of various factors.

Over the period spanning from 2001 to 2030, the GSCI demonstrates a consistent downward trend in both scenarios, albeit at a faster pace under the scenario with the R–U conflict (−0.014/(10a)) compared to the scenario without the conflict (−0.002/(10a)). These findings emphasize the significant disruptive effect of the external shock generated by the Russia–Ukraine conflict on the overarching evolutionary pattern of GSCI throughout the 30-year period. When comparing the projected results (2023 −2030 year) between the two scenarios, it becomes evident that the amplitude of GSCI fluctuations is greater under the scenario with the R–U conflict, ranging from 0.356 to 0.615, as opposed to the narrower range of 0.485 to 0.528 observed under the scenario without the conflict. This disparity underscores the substantial impact of the Russia–Ukraine conflict on the prospective global grain security landscape.

It can thus be seen that the R–U conflict still poses a serious risk to future global grain security and exerts serious pressure on the achievement of the 2030 SDGs, requiring a series of countermeasures by governments and international organizations [62,63]. At the same time, the global situation is still very unstable, which has brought considerable uncertainty to the already tense global food market. War conflicts show us again that the suffering caused by the effects of conflicts goes far beyond the battlefield. Russia’s war of aggression against Ukraine is exacerbating the global food crisis. The world and countries attempt to improve food and nutrition security to prevent social conflict and new wars [10,13].

## 5. Discussion

Against the backdrop of global climate change, armed conflicts serve as significant drivers exacerbating food insecurity [2,7,10]. Geopolitical conflicts not only affect the ability of food-producing or food-importing nations to maintain the normal functioning of their food systems and supply chains but also impede consumers’ access to an adequate food supply [10,11]. These two aspects reflect the availability and access dimensions of the GSCI. The R–U conflict is one of the notable global crises in recent years [13], with significant implications for global food supply chains, food prices, and food market trade [24,64]. This is primarily due to the fact that Russia and Ukraine are major food-producing and food-exporting nations, holding significant importance in the global food market [24].

This study emphasizes the global significance of Russia and Ukraine as agricultural powerhouses and major grain exporters. Both countries possess substantial grain reserves and have long played a crucial role in supplying grains, particularly wheat, barley, and corn, to numerous underdeveloped and food-deficient countries. These countries include regions such as the Middle East, North Africa, West Asia, Central Asia, as well as specific nations like Egypt, Turkey, Lebanon, and Yemen, which exhibit significant demand for grain imports. Notably, a substantial portion of Egypt’s wheat imports, around 90%, is sourced from Russia and Ukraine, while over 30% of Turkey’s corn imports originate from Russia [16]. On one hand, this highlights that the fluctuations in grain production in Russia and Ukraine serve as the primary driving force behind the uncertainty observed in the global grain market. On the other hand, it confirms that the ongoing conflict between Russia and Ukraine will disrupt the global food supply chain, resulting in severe consequences for the food security of importing nations. The disruption poses significant risks of food shortages and even famine in these countries. This situation arises from the inability of Russia and Ukraine to meet the grain demands and supplies of importers during the period of conflict.

This study provides compelling evidence, using a range of grain security indicators encompassing availability, access, and stability, to demonstrate the significant and predominantly negative impact of the Russia–Ukraine conflict on current and future global grain security. The conflict has resulted in a multitude of direct and indirect consequences across various dimensions of grain security. Firstly, with regards to grain supply and stability, the war has inflicted extensive damage upon farmland, irrigation systems, and infrastructure, thereby impeding the agricultural activities of farmers. The enlistment of soldiers and the displacement of populations have led to an acute shortage of agricultural labor, profoundly undermining agricultural production and diminishing a nation’s agricultural capacity. Consequently, there exists a severe deficit in both domestic and international food supply [24,65]. This situation poses immediate and medium-term ramifications for local communities and economies reliant on food exports, including the risk of food shortages and even famine. Secondly, in terms of grain accessibility, the conflict has exerted both short-term and long-term effects on the transportation of agricultural products within and beyond the borders of Ukraine. Specifically, the destruction of port facilities and railway systems has severely hampered the transportation of grain from Ukraine [64]. Prominent ports such as Odessa, Mariupol, and Kherson, responsible for over 90% of Ukraine’s grain and agricultural exports, have incurred substantial damage as a result of the war. Moreover, the blockade of Black Sea ports has significantly impeded Ukraine’s external grain trade [12]. Consequently, this situation poses a grave threat to consumers reliant on grain imports from Ukrainian ports, as they are unable to access a sufficient and timely food supply to meet their dietary requirements and ensure their survival.

Overall, there is continued uncertainty due to the Russia–Ukraine conflict, and the global food crisis is clearly not over. Food security is related to national security, human security, and global sustainable development. To cope with the global food crisis, governments and international organizations should actively take actions. Each country needs to reassess the risks and difficulties of its own national food security from multiple dimensions of food security, and restructure and improve its own food system so that it remains resilient in the long run and ensures food security in the face of rising climatic, conflict-related, and economic risks. The international community needs to build a sustainable global food security architecture based on sustainable development goals. Only in this way can we ensure the survival of all humankind so that human security is the basic guarantee.

This paper explores the overall level of global grain security only from a macro global perspective, and provides global views and macro ideas for the in-depth exploration of the spatial differences in food security risks within various countries and regions. Moreover, multi-dimensional and multi-indicator grain security evaluation can provide a greater decision-making basis for formulating macro-level global food security policies than evaluation based on a single dimension or a single indicator. On the other hand, the global grain security composite index focuses on grain security, which is established from the perspective of macro conditions throughout the world, without fully consider the utilization of food by micro-individuals. In the future, incorporating government and micro-individual nutrition security into a comprehensive analytical framework will bring more insights into the development of food security strategies.

## 6. Conclusions

This paper aimed to construct a grain security composite index to assess global grain security. The ARIMA model was employed to predict the future development of the global grain security composite index until 2030. By comparing and analyzing the grain security changes under scenarios with and without the “Russia-Ukraine conflict”, the potential impact of the conflict on future global grain security was revealed. The main conclusions are as follows:

(1) Russia and Ukraine have an important position in global food supply and trade, and in food markets. The two countries are not only major grain producers of wheat, barley, and corn but also important grain exporters. In 2023, Russia is the world’s fourth largest wheat producer and the largest wheat exporter, the tenth largest corn producer and the sixth largest corn exporter, and the second largest barley producer and the third largest barley exporter. Ukraine is the world’s eleventh largest wheat producer and seventh largest exporter, the eighth largest corn producer and fourth largest exporter, and the seventh largest barley producer and the sixth largest barley exporter.
(2) Global food prices have reached a record high due to the impact of the Russia–Ukraine conflict. Under the continuous impact of the conflict, food prices show a fluctuating trend of first increasing and then decreasing, and wheat price has increased the most. This may increase the uncertainty of the global food market and may have serious consequences for global food security.
(3) The conflict between Russia and Ukraine had a negative impact on global grain security. Global grain security showed an upward trend during the period without the R–U conflict (2001–2021) but a downward trend during the period with the R–U conflict (2001–2022). It is expected that by 2030, the global grain security level will show a trend of first decreasing and then increasing with and without the R–U conflict scenarios, but the change will be greater with the R–U conflict scenario. These results conclude that the future of global grain security will be affected by the continued impact of the conflict between Russia and Ukraine, and the prospects for achieving the 2030 SDGs are more worrisome.

The ongoing Russia–Ukraine conflict represents a significant emergency that has emerged after the COVID-19 pandemic, posing a tremendous impact on the global food system and international markets. At a time when the global economic and food insecurity situations are still in the process of recovering from the devastating consequences of the pandemic, the escalating conflict between Russia and Ukraine has the potential to exacerbate global instability and increase the likelihood of significant “cascade effects” or “risk cascades” on global socio-economic development and sustainability. In an increasingly complex and uncertain world, the food system is under mounting pressure to ensure an adequate food supply for the global population and mitigate the effects of intricate global changes on food production. As such, governments, international institutions, the private sector, and civil society organizations worldwide need to strengthen their efforts to bolster food production capacity, ensure food security, and establish a comprehensive and effective monitoring, and early warning mechanism for grain security, enabling them to proactively prevent and respond to major emergencies. However, addressing these challenges necessitates confronting numerous technological difficulties and obstacles, thereby requiring interdisciplinary research and innovative solutions at the intersecting frontiers. Therefore, this study aims to construct an indicator system for evaluating grain security and develop a grain security composite index (GSCI) from a global perspective. Additionally, it proposes a scenario-based comparative analysis approach employing econometric modeling to quantitatively forecast the potential impacts of emergencies on future grain security. By doing so, the study aims to offer scientific support for establishing a more resilient and sustainable food system.

## Figures and Tables

**Table 1:** Evaluation indicator system and measurement methods for grain security.

First-Layer Index	Second-Layer Index	Third-Layer Indicator (Unit)	Description	Type ^1^
Grain security composite index (GSCI)	$\mathrm{Availability}\;(Y_{1}$)	$X_{11}$: Gross per capita production index number	$X_{11}=$ production Index number/population index	positive
		$X_{12}$: Cereal yield (hg/ha)	Harvested production per unit of harvested area for crop products	positive
		$X_{13}$: Cereal import dependency ratio (%)	$X_{13}$ = (cereal imports − cereal exports)/(cereal production + cereal imports − cereal exports)	negative
		$X_{14}:$ Food loss (tonnes)	Amount of the commodity in question lost through wastage during the year at all stages between the level at which production is recorded and the household, i.e., storage and transportation.	negative
		$X_{15}$: Per capita food production value variability (thousand int$)	$X_{15}$ = standard deviation of the per capita food production value/average per capita food production value	negative
	$\mathrm{Access}\;(Y_{2}$)	$X_{21}$: Gross domestic product per capita ($)	$X_{21}=$ gross domestic product converted by purchasing power parity/total population	positive
		$X_{22}$: The agriculture orientation index for government expenditures	$X_{22}=$ share of agriculture in government expenditures/share of agriculture in GDP	positive
		$X_{23}:$ Value of food imports in total merchandise exports (%)	$X_{23}=$ value of food imports/total merchandise exports	negative
		$X_{24}$: The FAO cereal price index	a measure of the monthly change in international prices of a basket of food commodities	negative
		$X_{25}$: Food price inflation	fluctuation of grain commodity price series in a certain period	negative
		$X_{26}$: Rail line density (%)	the ratio between the length of railway routes available for train service	positive
	$\mathrm{Stability}\;(Y_{3}$)	$X_{31}$: Percentage of arable land equipped for irrigation (%)	ratio of the irrigated land area to the cultivated land area	positive
		$X_{32}$: Corruption index	the pervasiveness of corruption in a country by assessing the risk of corruption	negative
		$X_{33}:$ Urban absorption capacity	$X_{33}=$ average (annual) real percentage change in GDP − the urban population growth rate	positive

^1^ A positive indicator indicates a positive influence on grain security, meaning that the greater the value is, the higher the grain security level. A negative indicator indicates a negative influence on grain security, meaning that the greater the value is, the lower the grain security level.

**Table 2:** List of countries in the study area.

Number	Asia	Europe (EU)	Latin America and Caribbean (LAC)	Sub-Saharan Africa (SSA)
1	Azerbaijan	Austria	Argentina	Benin
2	Bangladesh	Belgium	Bolivia (Plurinational State of)	Botswana
3	China	Belarus	Brazil	Burkina Faso
4	India	Bulgaria	Chile	Democratic Republic of the Congo
5	Indonesia	Czechia	Colombia	Côte d’Ivoire
6	Israel	Denmark	Costa Rica	Ethiopia
7	Japan	Finland	Dominican Republic	Ghana
8	Jordan	France	Ecuador	Kenya
9	Kazakhstan	Germany	El Salvador	Madagascar
10	Kuwait	Greece	Guatemala	Malawi
11	Lebanon	Hungary	Honduras	Mali
12	Malaysia	Ireland	Mexico	Mozambique
13	Mongolia	Italy	Nicaragua	Niger
14	Nepal	Lithuania	Panama	Nigeria
15	Oman	Netherlands	Paraguay	Senegal
16	Pakistan	Norway	Peru	Sierra Leone
17	Philippines	Portugal	Uruguay	South Africa
18	Republic of Korea	Romania		Togo
19	Saudi Arabia	Russian Federation		Uganda
20	Sri Lanka	Slovakia		Zambia
21	Thailand	Spain		
22	Türkiye	Sweden		
23	Uzbekistan	Switzerland		
24	Viet Nam	Ukraine		
25		United Kingdom of Great Britain and Northern Ireland		

**Table 3:** Weight values of grain security indicators.

First-Layer Index	Second-Layer Index	Third-Layer Indicator	Weight
Grain security composite index (GSCI)	$\mathrm{Availability}\;(Y_{1}$)	$X_{11}$: Gross per capita production index number	0.047
		$X_{12}$: Cereal yield	0.045
		$X_{13}$: Cereal import dependency ratio	0.038
		$X_{14}:$ Food loss	0.107
		$X_{15}$: Per capita food production value variability	0.048
	$\mathrm{Access}\;(Y_{2}$)	$X_{21}$: Gross domestic product per capita	0.084
		$X_{22}$: The agriculture orientation index for government expenditures	0.043
		$X_{23}:$ Value of food imports in total merchandise exports	0.075
		$X_{24}$: The FAO cereal price index	0.249
		$X_{25}$: Food price inflation	0.100
		$X_{26}$: Rail line density	0.032
	$\mathrm{Stability}\;(Y_{3}$)	$X_{31}$: Percentage of arable land equipped for irrigation	0.053
		$X_{32}$: Corruption index	0.037
		$X_{33}:$ Urban absorption capacity	0.040

**Table 4:** Scenario assumptions for predicting the impact of the Russia–Ukraine conflict on future grain security.

Senario	Base Period	E	$X_{i}$	Y	∆Y
Without the R–U conflict	2001–2021	0	$X_{i1}$	$Y_{1}=f\left(0,\;X_{i1}\right)$	$∆Y=Y_{2}-Y_{1}=f\left(∆E,\;X_{i1}+∆X_{i}\right)-f(0,\;X_{i1}$)
With the R–U conflict	2001–2022	∆E	$X_{i1}+∆X_{i}$	$Y_{2}=f\left(∆E,\;X_{i1}+∆X_{i}\right)$	

**Table 5:** Unit root test of time series of the grain security composite index.

Scenario	Difference Order	*T*-Test Value	*p*-Value	AIC	Critical Value		
					1%	5%	10%
With the “Russia-Ukraine conflict” scenario (2000–2022)	0	−4.854	0.000 ***	−48.613	−3.964	−3.085	−2.682
	1	−1.894	0.335	−44.975	−4.223	−3.189	−2.73
	2	−0.188	0.940	−32.951	−4.332	−3.233	−2.749
Without the “Russia-Ukraine conflict” scenario (2000–2021)	0	−2.476	0.121	−54.599	−4.223	−3.189	−2.73
	1	−1.34	0.611	−39.153	−4.332	−3.233	−2.749
	2	−3.574	0.006 ***	−35.925	−3.924	−3.068	−2.674

Note: *** represents the significance level of 1%.

**Figure A1:**
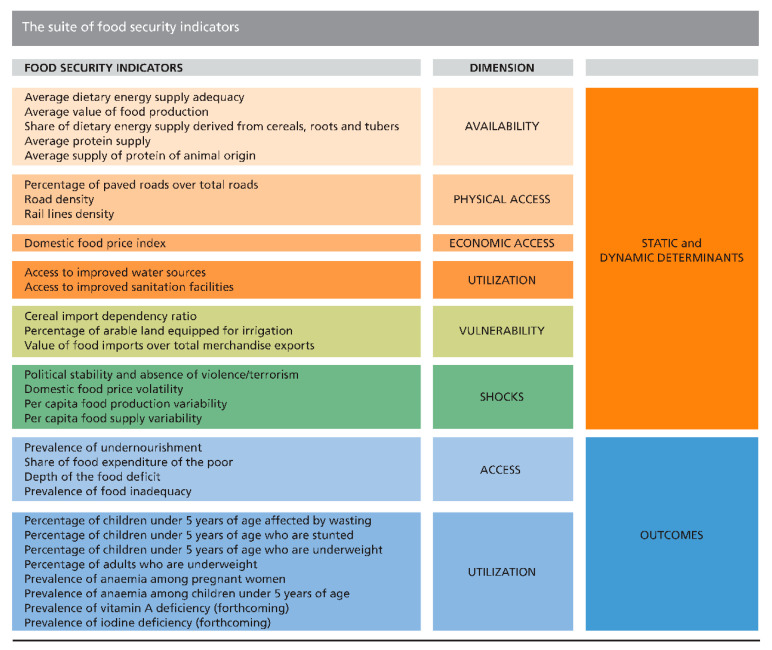
FAO’s food security indicator system [22].

**Figure A2:**
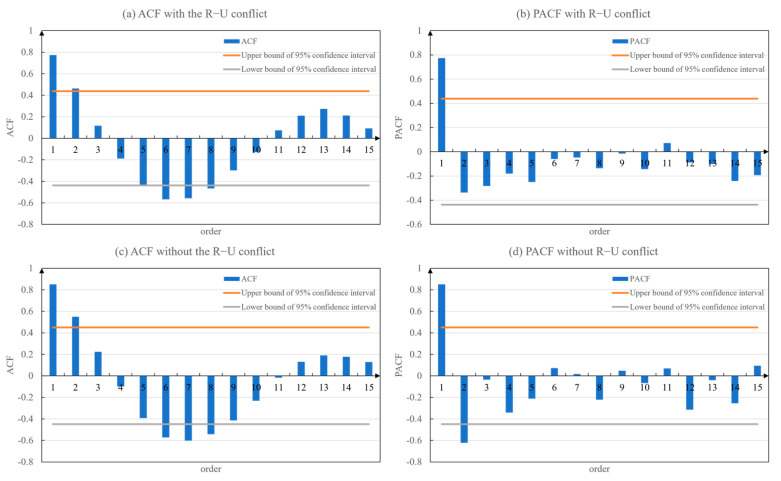
The autocorrelation and partial autocorrelation tests for the global grain security composite index (GSCI) under the scenarios “with” and “without” the Russia–Ukraine conflict.

**Figure A3:**
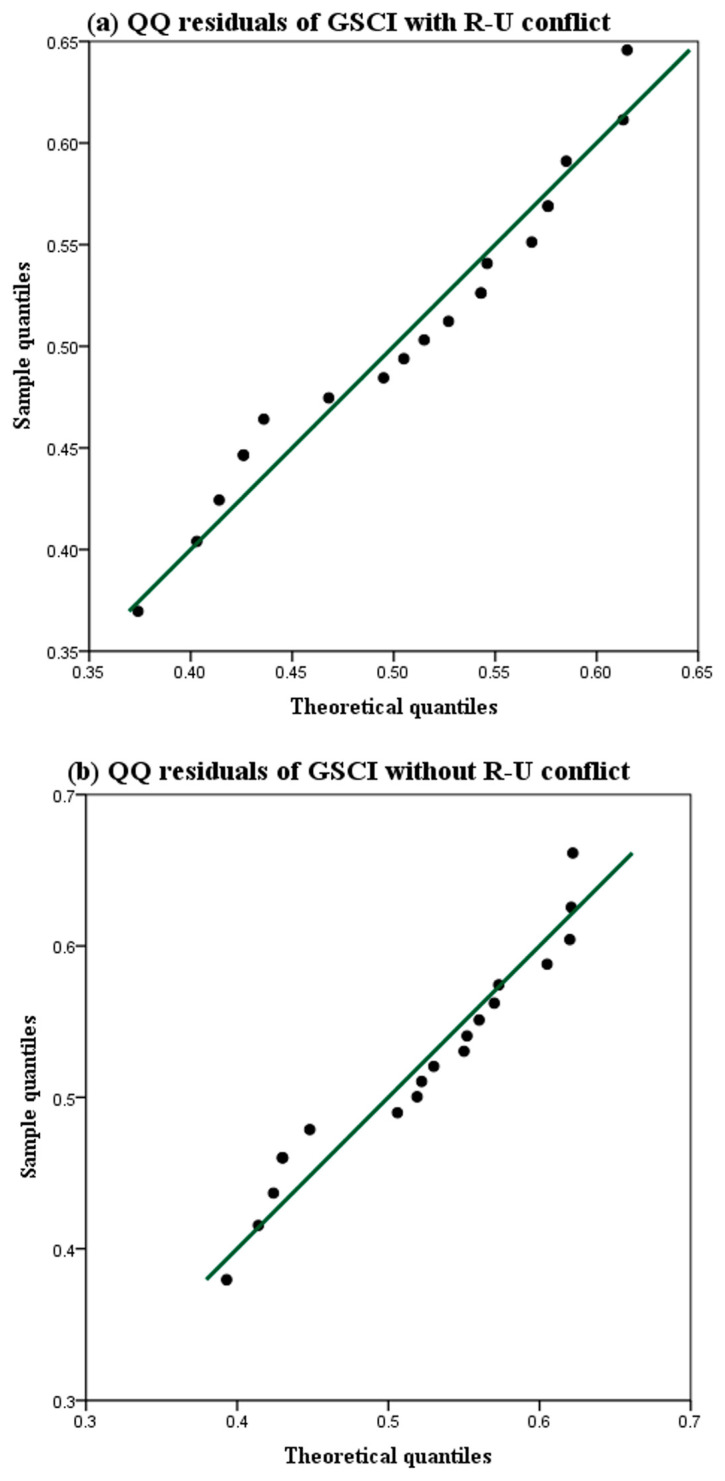
Residual chart of QQ model for GSCI predicted by ARIMA model (2001–2022).

**Figure A4:**
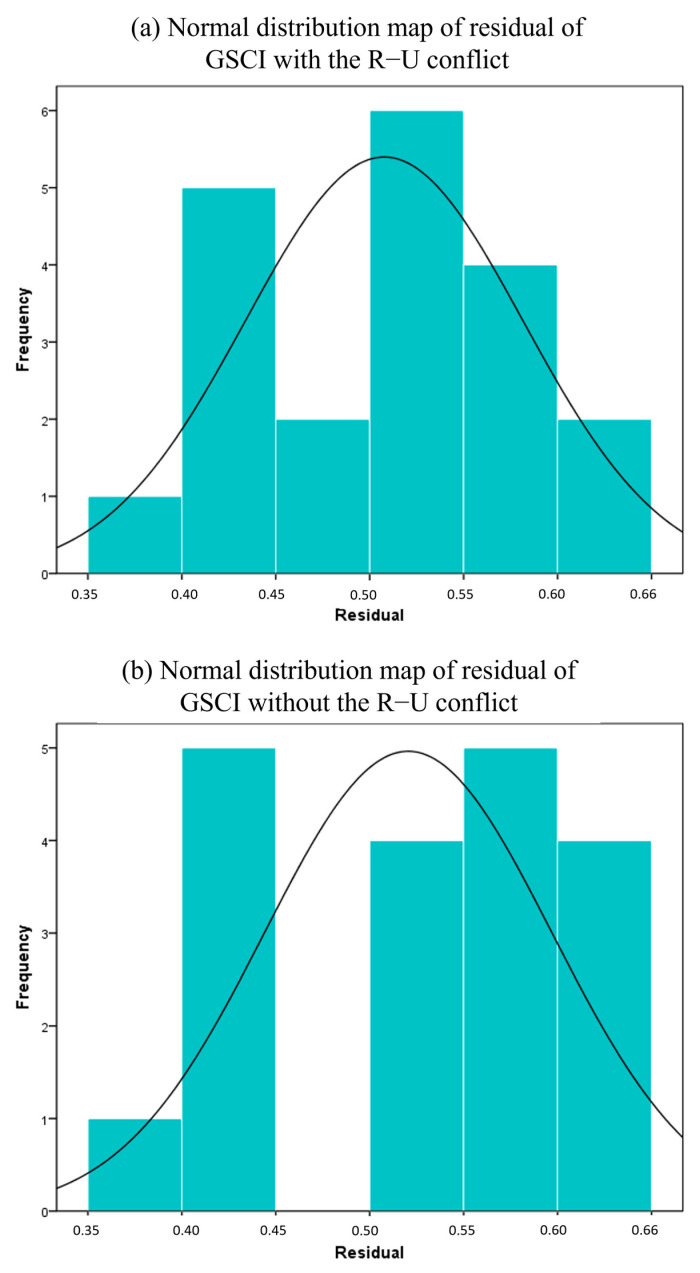
Normal distribution map of residual for GSCI predicted by ARIMA model (2001–2022).

## Data Availability

The datasets used and analyzed during the current study are available from the corresponding author upon reasonable request.

## Supplementary Materials

The following supporting information can be downloaded at: https://www.mdpi.com/article/10.3390/foods12132557/s1.
